# Environmental Regulation of Prions in Yeast

**DOI:** 10.1371/journal.ppat.1002973

**Published:** 2012-11-15

**Authors:** Liming Li, Anthony S. Kowal

**Affiliations:** Department of Molecular Pharmacology and Biological Chemistry, The Feinberg School of Medicine, Northwestern University, Chicago, Illinois, United States of America; Washington University School of Medicine, United States of America

## The Yeast Prion Concept

The term prion, proteinaceus infectious particle, was first used to describe the causative agent of a group of mammalian neurodegenerative diseases known as transmissible spongiform encephalopathies (TSEs) [1]. The mammalian prion protein (PrP) can exist in either a normal cellular conformation, PrP^C^, or in multiple misfolded pathogenic conformations, collectively called PrP^Sc^. PrP^Sc^ is considered infectious because it can recruit and convert its normal isomer PrP^C^ to its pathogenic conformation. This “protein-only” concept of infectivity has gained general acceptance and has been extended to explain some unusual non-Mendelian genetic elements in the budding yeast *Saccharomyces cerevisiae*. In yeast, these factorsare transmitted from mother to daughter cell as particular self-propagating protein conformations, and are thus referred to as yeast prions [2].

Yeast prions share many features with PrP^Sc^: both are capable of perpetuating particular conformational changes, forming amyloid fibrils (ordered protein aggregates with cross-β sheet structure and filamentous morphology) under physiological conditions, and both can exist as multiple “strains” or variants. However, a number of fundamental differences between them are worth noting. First, yeast prion proteins and PrP do not share a significant sequence similarity. Almost all yeast prion proteins contain a domain with an unusually high content of glutamine (Q) and asparagine (N) residues (∼45%), whereas PrP does not have such a region. The Q/N-rich domains of yeast prion proteins, termed prion forming domains (PrDs), are modular and transferrable and essential for the formation and propagation of their corresponding prions. Second, whereas the normal function of PrP is unclear, yeast prion proteins are involved in a wide range of functions, from transcriptional and translational regulation to nitrogen metabolism. To date, PrP is the only prion protein identified in mammals, whereas at least 8 prions have been identifiedin yeast: [*PSI* ^+^], [URE3], [*PIN* ^+^], [*SWI^+^*], [*OCT* ^+^], [*MOT3*], [*ISP* ^+^], and [*MOD^+^*] [3], [4] (capital letters indicate that these genetic elements are dominant, and brackets signify non-Mendelian patterns of inheritance). Finally, while PrP^Sc^ is associated with human disease, yeast prions are not associated with disease *per se*, but manifest as dominant, cytoplasmically inherited phenotypes.

## Protein-Based Infectivity of Yeast Prions

**Figure 1:**
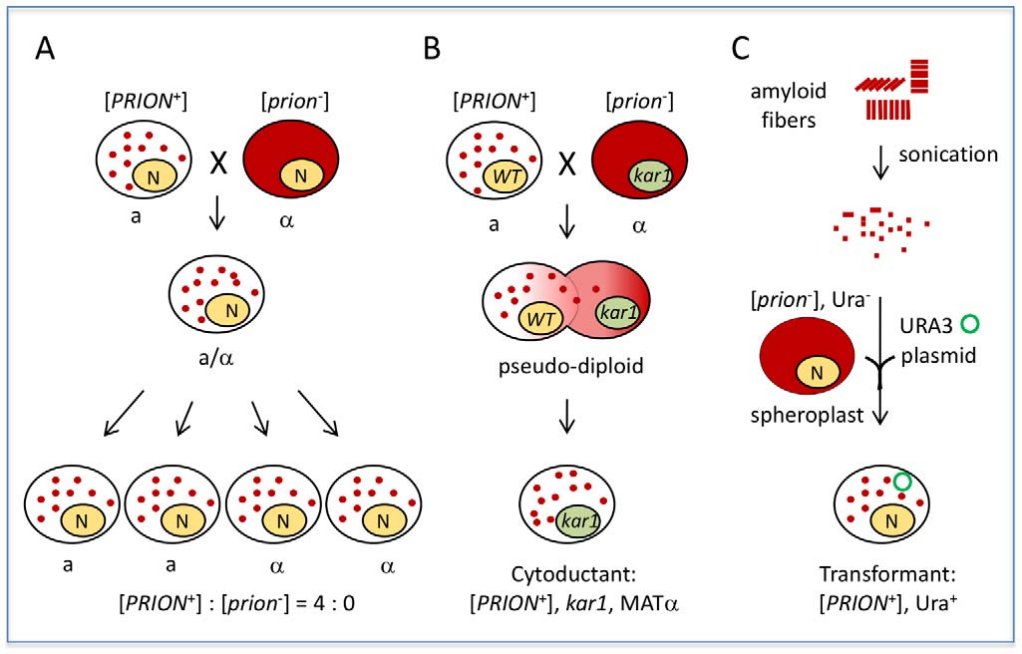
Yeast prions are “infectious.” A) A sexual cross of [*PRION* ^+^] and [*prion^−^*] cells of opposite mating types results in a [*PRION* ^+^] diploid, which can give rise to fourspores that are all [*PRION* ^+^] after sporulation. Note: in the case of weak [*PSI* ^+^] and [URE3], [*PRION* ^+^]×[*prion* ^−^] crosses do not always give a 4∶0 segregation in progeny. Some other random, non-Mendelian segregation ratios of progeny can be seen, such as 1∶4, 1∶3, 3∶1, 4∶0, as well as 2∶2, due to their meiotic instabilities. B) Mating a [*PRION* ^+^] donor with a [*prion* ^−^] recipient carrying a *kar1* mutation (which prevents nuclear fusion of the mating partners) will result in formation of a pseudodiploid carrying a mixed cytoplasm of the two mating partners. The pseudodiploid will give rise to haploid cytoductants containing either the donor or recipient nucleus. Shown is a cytoductant containing the recipient nucleus with a *kar1* mutation. C) Transformation of [*prion* ^−^] spheroplasts (yeast with cell wall removed) with amyloid fibers assembled from recombinant prion protein can result in *de novo* formation of heritable [*PRION* ^+^] in the transformed cells. A *URA3* plasmid (green circle) was used as a selection marker for the transformation. Solid red color indicates the soluble, diffused prion-determinant protein, whereas red dots indicate the prion protein is in an aggregated prion conformation.

Yeast prions do not infect nonprion cells through simple cell–cell contact. For example, coculturing [*PRION* ^+^] and [*prion* ^−^] cells of the same mating type does not result in prion transmission. However, sexual crosses between [*PRION* ^+^] and [*prion* ^−^] cells yield diploidsthat are all [*PRION* ^+^], and tetrads derived from [*PRION* ^+^] diploids will give rise to spores that are all [*PRION* ^+^] (Figure 1A). In contrast, a diploid from a similar cross of a nucleic acid–based mutant to a wild-type partner gives rise to meiotic progeny in a 2∶2 ratio. This “protein-only” infectivity of yeast prions can be also demonstrated by cytoduction, a process in which the cytoplasmic but not the nuclear components are mixed between partners (Figure 1B). [*PRION* ^+^] donor cells can pass the [*PRION* ^+^] state to nonprion recipient haploid progeny without exchange of genetic information. This “gold-standard” assay has been used to confirm if a phenotypic trait is cytoplamically inherited; all known yeast prions are cytoducible due to their protein-based infectivity. Prion infectivity can also be demonstrated by transformation of prion fibrils (Figure 1C). Incubating naïve [*prion* ^−^] cells with amyloid fibrils assembled *in vitro* from recombinant prion proteins can result in *de novo* formation of stable, transmissible prions in the recipient. The first successful studies to demonstrate fibril-based transformation were conducted using the well-studied prion [*PSI* ^+^], a translation termination modifier [5], [6]. This method of transformation provides simple, direct confirmation that the amyloidsformed *in vitro* are able to self-propagate by converting endogenously produced protein isomers into the [*PRION^+^*] state.

## An Interaction between Yeast Prions and the Cellular Machinery

While infectious prion amyloids can be formed in a test tube autocatalytically, prion formation and propagation inside a cell requiresa supporting cellular network; imbalance of this network often results in prion destabilization or loss. For example, inhibiting the activity of the protein deaggregase Hsp104, which normally fragments prion fibrils into transmissible seeds, blocks prion transmission from mother to daughter during cell division and results in the loss of all amyloid prions [7]. Further, the abundant yeast cytoplasmic chaperone (Hsp70-Ssa) collaborates with two groups of cochaperones—the Jprotein family members (e.g., Sis1) and the nucleotide-exchangefactors (e.g., Sse1)—to play a crucial role in maintaining yeast prions [8]. In prions that have been examined thus far, manipulating the function of Hsp70-Ssa or its cochaperones has been found to result in their destabilization or loss [8]. Other cellular factors thathave been identified as supporting the prionogenic cellular network include components of the cytoskeleton, the endocytotic machinery, and the ubiquitin-proteasome system (UPS) [9], [10]. Remarkably, a single yeast cell can harbor multiple prion elementssimultaneously, but they do not simply coexist; they can promote or inhibit each other's appearance and maintenance. For example, the presence of [*PIN* ^+^] or [URE3] can facilitate [*PSI* ^+^] induction [11]. However, they have also been shown to have antagonizing effects [11], [12]. Therefore, stable prion transmission is a consequence not only of dynamic interactions between coexisting prions and their protein determinants but also of other cellular components.

## Environmental Regulation of Yeast Prions

**Figure 2:**
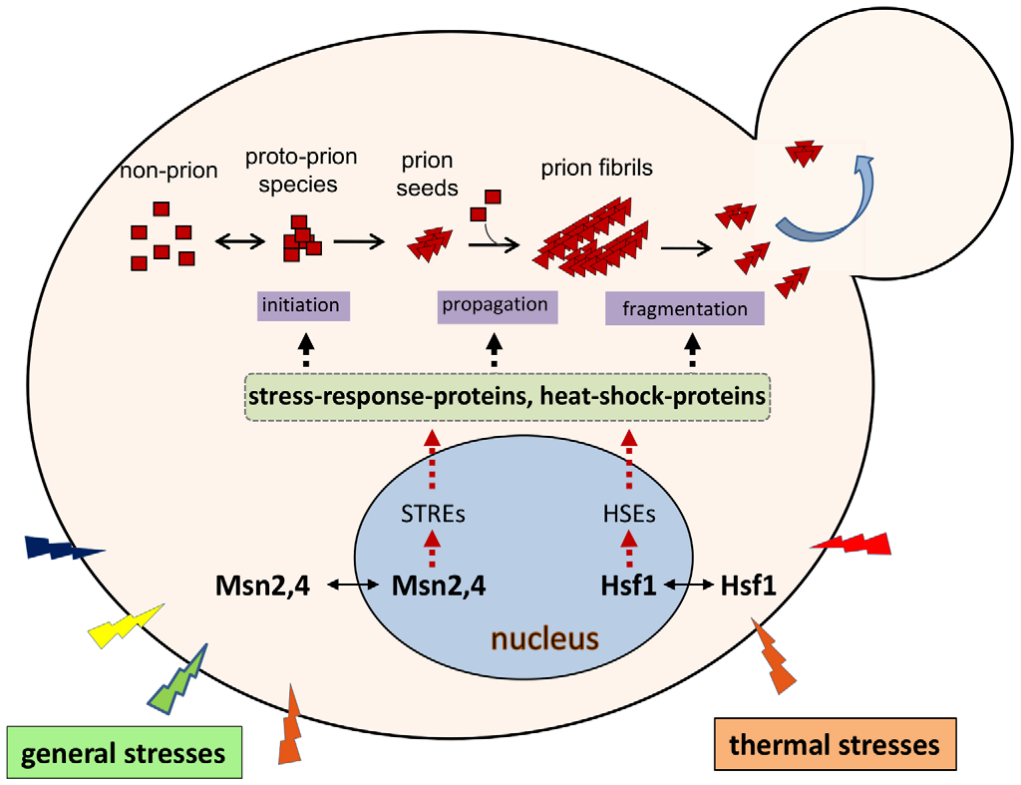
Environmental regulation of yeast prions. Prionogenesis is a multistep process in which the prion determinant protein undergoes changes in its secondary structure to form intermediate species and then prion (amyloid) fibrils; this process relies on other cellular machinery to drive these changes. Thermal stress results in the relocalization of heat-shock factor 1 (Hsf1) from the cytoplasm to the nucleus; here it binds to the heat-shockelement (HSEs) of heat-shock–protein genes, activating their transcription. Consequentially, a diverse group of heat-shock proteins (HSPs) are synthesized. Many HSPs (molecular chaperones) play important roles in prion formation and propagation, including Hsp104, Hsp70-Ssa, and Hsp40-Sis1. In a similar manner, general stresses including oxidative, osmotic, and heat stresses, activate a separate pathway in which Msn2,4 binds to the stress-response element (STREs) of stress-response genes, thereby activating their transcription. Some HSP genes also contain one or more STREs at their 5′-regulatory regions. Deletion of the *MSN2* gene results in a drastic increase of the frequency of [*PSI* ^+^] formation, suggesting that some stress-response proteins are also involved in prion formation. However, the identity of the Msn2,4 targets that are involved in prionogenesis remain elusive. Note: for simplicity, only the two major stress-response pathways that are regulated by Hsf1 and Msn2,4 are shown.

Prion proteins interact extensively with their cellular environments throughout the entire process of prion formation and propagation (Figure 2). Therefore any modulations that perturb this cellular interaction network will likely affect prionogenesis and prion stability. Intriguingly, supplementation of growth media with select chemical agents, such as the protein denaturant guanidine hydrochloride, the organic solvent dimethyl sulfoxide, alcohols, or potassium chloride salt, results in the loss or destabilization of the [*PSI* ^+^] prion [13]. Thermal changes, treatments with antibiotics, or oxidative chemicalsalso have profound effects on [*PSI* ^+^] propagation [13]–[15]. Prion *de novo* formation can be affected by environmental stresses as well. Mutations in heat-shock factor 1 (Hsf1), the master regulator of heat-responsegenes, drastically influence the frequency of [*PSI* ^+^] induction. The observed effects of [*PSI* ^+^] induction can be either an enhancement or inhibition, depending on the specific nature of the Hsf1 mutation [16]. In addition, data from an unbiased, high-throughput screen identified a group of stress-response proteins, including Msn2, a general stress-response regulator, and Hac1, a protein-unfolding response regulator,as modifiers of [*PSI* ^+^] prionogenesis [17]. Mutants harboring deletion of *MSN2* or *HAC1*, or the exposure of wild-type cells to various extreme stressful conditions, drastically increased the frequency of [*PSI* ^+^] induction [17]. Recent findings show that heatshock increases the synthesis of Lsb2, a short-lived protein facilitating [*PSI* ^+^] *de novo* formation [9], suggesting another regulatory mechanism for the impact of environment on yeast prionogenesis.

## Potentially Diverse Roles for Yeast Prions in Evolution

Isogenic [*PRION* ^+^] and [*prion* ^−^] cells may exhibit completely different phenotypes under identical environmental conditions, but they can switch between these distinct phenotypic states spontaneously. It has been proposed that prion formation may be a mechanism to uncover otherwise hidden genetic variations to create new phenotypic traits, thus providing a means of rapid adaptive evolution [14], [18]. Indeed, the metastable nature of prion inheritance offers a potential for regulatory plasticity that cannot be readily achieved by nucleic acid mutation. Because prion-conferred phenotypic traits can be quickly spread between mating partners and progeny without altering the underlying nucleic acid sequence, prion-based inheritance might provide a rapid means to allow yeast to survive sudden undesirable environmental changes. That yeast prions and mammalian PrP^Sc^ can exist as multiple heritable variants indicates the possibility of multilevel epigenetic regulation. Additionally, in its aggregated conformation, a prion protein may sequester other important cellular factors, causing, in effect, a multigene-knockdown phenotype. Lastly, since a single yeast cell can harbor multiple prion elements simultaneously, it is possible that different prion combinations might provide additional phenotypic diversity.

Indeed, it has been hypothesized that the [*PSI* ^+^] prion aidsthe response of yeast to environmental changes in order to produce a number of new, temporary phenotypic traits [14]. Remarkably, some [*PSI* ^+^]-mediated epigenetic traits can be fixed permanently in progeny through one-step outcross to become [*PSI* ^+^] independent [18]. While it remains controversial whether the presence of a prion is beneficial to yeast [19], recent studies provide evidence to support the hypothesis that the prions provide a fitness advantage. For example, the recently discovered prion [*MOD* ^+^] confers a gain-of-function resistance to antifungal agents [4]. Upon application of antifungal drugs, [*MOD^+^*] prion conversion increases, suggesting that *de novo* prion appearance is effected by selective pressure [4]. Crucially, yeast prions are not an artifact of laboratory manipulation; a recent study found several yeast prions ([*PSI* ^+^], [*PIN* ^+^], and [*MOT3* ^+^]) in a number of wild strains [20], indicating that these prions arise from some selective pressure under natural conditions. Collectively, prion-mediated heritable conformational alterations potentiate evolutionary changes.

## Conclusions

Although yeast prions are not associated with distinct human diseases, results from yeast prion research during the last two decades have provided invaluable information about protein misfolding, aggregation, and protein-based heredity and infectivity. The fact that multiple prions have been identified in yeast thus far (with additional promising prion candidates) suggests that their occurrence is a ubiquitous, natural biological phenomenon that deserves our understanding and further research efforts. Due to its simplicity and amenability to genetic and cell biological manipulation, yeast will remain a powerful model organism for prion research.
